# Iconic Arable Weeds: The Significance of Corn Poppy (*Papaver rhoeas*), Cornflower (*Centaurea cyanus*), and Field Larkspur (*Delphinium consolida*) in Hungarian Ethnobotanical and Cultural Heritage

**DOI:** 10.3390/plants12010084

**Published:** 2022-12-23

**Authors:** Gyula Pinke, Viktória Kapcsándi, Bálint Czúcz

**Affiliations:** 1 Albert Kázmér Faculty of Mosomagyaróvár, Széchenyi István University, Vár 2., H-9200 Mosonmagyaróvár, Hungary; 2 European Commission, Joint Research Centre, Via Fermi 2749, 21027 Ispra, Italy

**Keywords:** anthropology, arable weed conservation, charismatic species, cultural history, cultural symbols, ethnobotany, human–plants relations, medicinal plants, wild food plants

## Abstract

There are an increasing number of initiatives that recognize arable weed species as an important component of agricultural biodiversity. Such initiatives often focus on declining species that were once abundant and are still well known, but the ethnographic relevance of such species receives little recognition. We carried out an extensive literature review on the medicinal, ornamental, and cultural applications of three selected species, *Papaver rhoeas*, *Centaurea cyanus,* and *Delphinium consolida*, in the relevant Hungarian literature published between 1578 and 2021. We found a great diversity of medicinal usages. While *P. rhoeas* stands out with its sedative influence, *D. consolida* was mainly employed to stop bleeding, and *C. cyanus* was most frequently used to cure eye inflammation. The buds of *P. rhoeas* were sporadically eaten and its petals were used as a food dye. All species fulfilled ornamental purposes, either as garden plants or gathered in the wild for bouquets. They were essential elements of harvest festivals and religious festivities, particularly in Corpus Christi processions. *P. rhoeas* was also a part of several children’s games. These wildflowers were regularly depicted in traditional Hungarian folk art. In poetry, *P. rhoeas* was used as a symbol of burning love or impermanence; *C. cyanus* was frequently associated with tenderness and faithfulness; while *D. consolida* regularly emerged as a nostalgic remembrance of the disappearing rural lifestyle. These plants were also used as patriotic symbols in illustrations for faithfulness, loyalty, or homesickness. Our results highlight the deep and prevalent embeddedness of the three iconic weed species studied in the folk culture of the Carpathian Basin. The ethnobotanical and cultural embeddedness of arable weed species should also be considered when efforts and instruments for the conservation of arable weed communities are designed.

## 1. Introduction

Farmers and agronomists have been desperately engaged in reducing the adverse economic effects of arable weeds for a long time. Nevertheless, arable weeds may also exhibit beneficial properties [1,2] and they can also contribute to several important ecosystem services, for example, pest control and soil fertility improvement [3,4,5,6]. Moreover, weeds are often the basis of agricultural food webs providing food resources to many organisms, including numerous insect and bird species, so they are considered beneficial from a conservation or even from an agricultural point of view [7,8,9]. Weeds, which exhibit a low level of competition with crops and provide a considerable resource value for higher trophic groups, are sometimes distinguished as “good weeds” [10,11]. Several of these “good” arable weed species have become threatened by agricultural intensification in Europe [12,13,14]. The decline of well-known, often colourful “emblematic”, weed species has been recognized in several EU-level conservation initiatives, including arable plant sanctuaries [15,16]. Nevertheless, these iconic species are also deeply embedded in the local culture of the European rural regions and are accompanied by considerable traditional knowledge, and ethnobotanical and cultural heritage, which is also becoming endangered with their decline [17,18,19]. In turn, the regional cultural embeddedness of these species should also be considered as an important factor in the design of the conservation programmes that are aimed at protecting arable weed communities for the future generations.

In this paper, we aim to explore the cultural embeddedness of three emblematic arable weed species: corn poppy (*Papaver rhoeas* L., henceforward *poppy*), cornflower (*Centaurea cyanus* L., syn. *Cyanus segetum* Hill), and field larkspur (*Delphinium consolida* L., syn. *Consolida regalis* Gray, henceforward *larkspur*), in the culture of Hungarian-speaking communities in the Carpathian Basin in Eastern Europe from a diachronic perspective.

**Figure 1:**
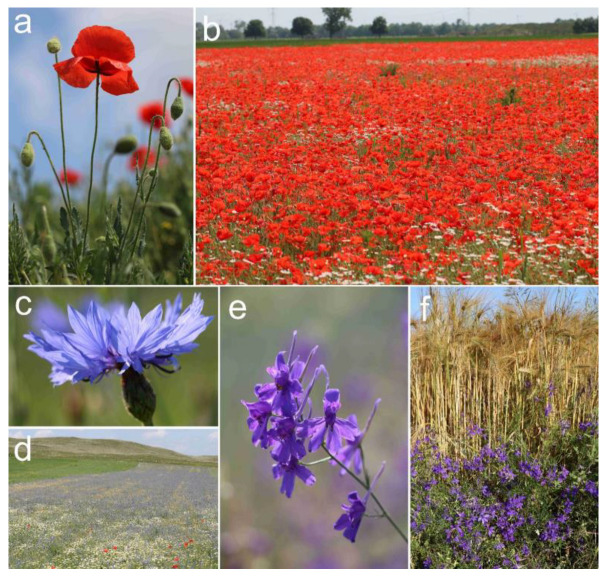
The three studied weed species and their spectacular mass occurrences in arable fields: (**a**,**b**) *Papaver rhoeas* (Hegyeshalom, NW-Hungary, 2018); (**c**,**d**) *Centaurea cyanus* (Öskü, W-Hungary, 2011); (**e**,**f**) *Delphinium consolida* (Püski, NW-Hungary, 2020; all photographs by Gyula Pinke).

Although sacred plants [20], magical herbs [21,22], ritual [23] and long-lived [24] trees, antique fruits [25], peculiar food plants [26], and orchids [27,28] are frequently subjects of ethno-cultural botanical studies, very few studies focus on arable weed species with cultural significance [17,18,19]. The three weed species in the focus of this paper were introduced to Central Europe as archaeophytes [29] and their remains were found in archaeological sites, also in the Carpathian Basin, from the Copper Age until early modern times [30,31,32]. Former field observations [33], as well as contemporaneous findings, in adobe bricks [34] suggest that the three studied species were among the most abundant arable weeds by the end of the 19th century in the Carpathian Basin. Due to their brightly coloured flowers, all three species were popular wildflowers, and, despite their recent decline, they are still well known by the general public [35,36,37,38] (Figure 1). Because of their general recognition and charismatic nature, they could function as potential “flagship species” in conservation programmes [39] to combat the general decline of botanical interest and awareness (also known as “plant blindness” [40]), underlying many further recent global challenges [41].

The poppy is still relatively frequent in Central Europe, probably due to its persistent seed bank [42], and can be a noxious weed in some crops, including the opium poppy (*Papaver somniferum* L.) [43] or in regions where herbicide-resistant poppy biotypes have recently been detected [44]. The cornflower was once common in many European countries, but it has largely declined and become threatened almost everywhere due to agricultural intensification [12]. Now, it is considered as an indicator species of low input cereal fields; thus, it is often addressed as a “flagship species” in conservation programmes [45,46]. The larkspur was previously widespread and is now regionally rare in some European countries [47]. It functions as an emblematic species for reintroduction projects of rare arable plants [48]. Currently, in the central part of the Carpathian Basin, the cornflower has only sporadic distribution and is still decreasing, while the larkspur is still frequent [49].

In order to gather and document information about the cultural embeddedness of the three studied species, we performed an extensive literature review, focusing on historical sources available in the Hungarian language, with which we aimed to create a comprehensive inventory of the occurrence of these species in traditional folk culture, including medicinal, nutritional, and ornamental applications, as well as their cultural roles in traditional festivals and children’s games. Furthermore, we complement this overview with an outlook on the representation of these species in the visual arts and literature, providing further illustrations of the symbolic significance of these species. By doing so, we are opening up the “footprint” of these iconic weed species in Hungarian culture to a broad international audience, thus making this otherwise relatively inaccessible rich cultural heritage more accessible. We also hope that a better knowledge of this threatened cultural heritage can help improve and enrich the predominantly negative public discourse on arable weeds. Accordingly, to assign a weed as “beneficial” not only will its rarity status and importance in food chains be taken into account, but also its ethnobotanical and cultural relevance will be considered.

## 2. Methods

We performed a series of targeted literature searches in several Hungarian online databases, including Arcanum, Hungaricana Közgyűjteményi Portál, Matarka, Magyar Elektronikus Könyvtár, Elektronikus Periodika Adatbázis Archívum, and Erdélyi Magyar Elektonikus Könyvtár. These databases only contain works in Hungarian; accordingly, our study did not cover sources written in other languages of the Carpathian Basin. However, Hungarian articles that describe other ethnic groups living in the same area were included. As keywords, we used the names of the species in several forms: for scientific names we used the Plants of the World Online (POWO) database [50] as our primary reference (including also the main synonyms—see Introduction), whereas for Hungarian vernacular names (including regional and local folk names), we relied on the books of Wagner [33], Vörös [51], and Rácz [52] [i.e., *pipacs*, *pipats*, *pippancs*, *pipanc*, *papics*, *papantz*, *papcsik*, *pipók*, *vadmák*, *veres mák*, *lúdmák*, *czúczik*, *cucik*, *pitypalatyvirág* for *poppy*; *búzavirág*, *dődike*, *égi virág*, *kék virág*, *csüküllő*, *sukollat*, *vadpézsma*, *kékkonkoly*, *gabonavirág* for *cornflower*; and *szarkaláb*, *királyvirág*, *sarkvirág*, *sarkantyúfű*, *sarkasfű*, *dalisarkanytú*, *vitézi farkanytú* for *larkspur*]. We combined the species names with further search terms identifying possible cultural uses (e.g., the Hungarian terms for “ethnobotany”, “medicinal”, “remedy”, “edible”, “food”, “fodder”, “dye”, “ornamental”, “bouquet”, “wreath”, “garden”, “toy”, “game”, “festival”, “religion”, “feast”, “harvest”, “folklore”, “belief”, “symbolism”, “art”, “motif”, “handicraft”, “painting”, and “poetry”). For the literature databases which made this possible, we also extended the search to the whole text of the primary studies, and not just the title, abstract, and keyword fields. The studied historical sources are presented in Table 1, Table 2 and Table 3.

In a second step, our search expressions were translated into English, and we repeated the search in four selected major scientific literature databases (Web of Science, Scopus, Google Scholar, and ResearchGate). With this follow-up search, we aimed to place our results into a broader European context.

Most of the results are presented in a narrative format, but in the case of a few complex subtopics, that were particularly interesting and rich in details (medicinal uses, religious uses, and symbolic connotations related to human characters and feelings), we constructed tables to enumerate the results in a more structured form. For the interpretation of the archaic Hungarian names of diverse ailments, we used the book of Magyary-Kossa [53]. To present plant parts and modes of preparations, we followed the terminology of the American Botanical council [54].

In order to find relevant illustrations for our results, we made further ad-hoc searches on the websites of several Hungarian museums, other institutes, and online collections, applying the scientific and vernacular names of the target species as the main search terms. For some cultural uses without available original images, we created our own illustrations by reconstructing “animated scenes” depicting the activities (e.g., for children’s games) or by using related contemporary items (e.g., for medicinal and food dying uses).

**Table 1:** Records on the medicinal and veterinary uses of *Papaver rhoeas*, *Centaurea cyanus,* and *Delphinium consolida* between 1578 and 2018 in the Carpathian Basin.

Year of Publication/ Relevant Period	Source	Region (Current Country)	Species	Part Used	Mode of Preparation/ Administration	Treated Disease(s)/Folk Medical Use(s)
1578	Melius Juhász Péter [55]	Hungary	*P. rhoeas*	Fructus (“Poppy heads”)	Decoction (made with water or wine)/oral	Insomnia
				Not specified	Infusion/topical (mouthwash)	Mouth and gum diseases
					Infusion, poultice/topical (genitals)	Heavy menstruation bleeding
				Semen	Infused honey/oral	Intestinal pain
				Latex	Oral	Fever, throat, and tongue swelling
					Topical	“St Anthony’s fire” (erysipelas)
					Poultice/topical	Nose- and liver-bleeding
1595	Beythe András [56]	Hungary	*P. rhoeas*			*Same as Melius Juhász (1578)*
1690	Pápai Páriz Ferencz [57]	Hungary	*P. rhoeas*	Latex	Oral	Insomnia (particularly after venesection), stomach pain, dysentery
				Flos, latex	Oral	Bleeding
Early 18th c.	Unknown physician [58]	Transylvania (Romania)	*D. consolida*	Herba	Infused vinegar/topical (nose)	Nose-bleeding
18–19th c.	Gulyás Éva [59]	East Hungary	*P. rhoeas*	Flos	Infusion/oral	Breast pain
18–19th c.	Novák László [60]	East Hungary	*C. cyanus*	Flos	Infused wine, poultice/topical	Eye inflammation
			*P. rhoeas*	Not specified	Oral	Stomach pain
1775	Csapó József [61]	Hungary	*P. rhoeas*	Flos	Infusion/oral	Catarrh, pleurisy
			*C. cyanus*	Flos	Poultice/topical	Eye inflammation
			*D. consolida*	Herba	Powder/oral	Heartburn
				Green herba	Pressed sap/topical (washing and bandage)	Fresh wounds
				Flos	Decoction (in rose-water), poultice/topical	Eye inflammation
1789	Zsoldos Xavér [62]	West Hungary	*P. rhoeas*	Flos (petals)	Infusion/oral	Panacea
1798	Veszelszki Antal [63]	Hungary	*D. consolida*	Semen	Infused wine/oral	Plague, intestinal pain, lithiasis
				Herba	Decocted wine/oral	Parasitic worms
			*P. rhoeas*			*same as Melius Juhász (1578)*
			*C. cyanus*	Flos	Smashed powder/oral	Jaundice
					Poultice/topical	Eye inflammation
					Pressed sap/topical (mouthwash)	Bad breath
1813	Diószegi Sámuel [64]	Hungary	*P. rhoeas*	Flos (petals)	Infusion and syrup/oral	Pain relief
			*D. consolida*	Flos	Not specified	Parasitic worms, epilepsy
1899	Temesváry Rezső [65]	Hungary	*P. rhoeas*	Latex	Added to milk/oral	Gynaecological bleeding
				Not specified	Infusion/oral	Pain relief during childbirth
			*D. consolida*	Flos	Topical (hot bath)	Gynaecological bleeding
					Decoction added to red wine/oral	Gynaecological bleeding
					Fumigation/topical (vulva)	Premature birth prevention
				Not specified	Breast plaster, fumigation, poultice/topical	Mastitis
				Not specified	Infusion/oral	Pain relief during childbirth
1902	Gönczi Ferenc [66]	West Hungary	*D. consolida*	Flos	Decocted beer/oral	Bleeding
1910	Gönczi Ferenc [67]	West Hungary	*C. cyanus*	Not specified	Dew collected from the plant/topical (face wash)	Freckles
1925	Darvas Ferenc [68]	Hungary	*D. consolida*	Flos	Not specified	Conjunctivitis, chronic constipation, menstruation disorders
				Semen	Not specified	Lice and other skin parasites
			*C. cyanus*	Flos	Infusion (blend component)/oral	Colour enhancer
					Fumigant blends/topical	Colour enhancer
			*P. rhoeas*	Flos	Infusion/oral	Pain and spasm relief
					Not specified	Syrups, cough drops, dyeing sugar solutions
1928	Relkovic Davorka [69]	West Hungary	*D. consolida*	Flos	Not specified	Bleeding
1932	Rapaics Raymund [70]	Hungary	*P. rhoeas*	Fructus (“Poppy heads”)	Fresh poppy heads/oral (used as a “pacifier”)	Babies crying too much (sedative)
1935	Luby Margit [71]	Northeast Hungary	*D. consolida*	Flos	Decoction/topical (footbath) Dried flowers/used as a shoe insert	Bleeding
1940	Réthelyi József [72]	Hungary	*P. rhoeas*	Flos (petals)	Infusion/oral	Panacea, exorcism (to expel bad illness)
1941	Vajkai Aurél [73]	West Hungary	*D. consolida*	Not specified	Infusion/oral	Bleeding
				Not specified	Decoction (with horse chestnut)/oral	Haematuria (veterinary: cattle)
1944	Greszné Czimmer Anna [74]	East Hungary	*P. rhoeas*	Not specified	Infusion/oral	Heavy menstruation, gynaecological bleeding
			*D. consolida*	Flos	Infusion/oral	Bleeding after childbirth
1945	Vargyas Lajos [75]	Central Hungary	*C. cyanus*	Not specified	Infusion/oral	Cough
			*P. rhoeas*	Not specified	Decocted red wine/oral	Bleeding
				Flos	Decocted wine/oral	Inducing abortion
				Not specified	Oral	Contraception
			*D. consolida*	Not specified	Not specified	Contraception
1968	Farkas József [76]	Northeast Hungary	*D. consolida*	Not specified	Decoction/topical (hot bath) Shoe insert/topical	Bleeding
1969	Seregély György [77]		*P. rhoeas*	Not specified	Oral	Inducing abortion
1976	Péntek János [78]	Transylvania (Romania)	*C. cyanus*	Flos	Infusion/poultice	Eye inflammation
					Infusion/oral	Diuretic therapy
			*D. consolida*	Flos	Infusion	Leucorrhoea
					Tincture (in brandy)/oral	Bleeding
1976	Szabóné Futó Rózsa [79]	North Hungary	*D. consolida*	Herba	Decoction, poultice/topical (bath)	Eczema
			*P. rhoeas*	Not specified	Infusion/oral	Pulmonary diseases, cough, bleeding
			*C. cyanus*	Not specified	Infusion/oral	Pulmonary diseases, bleeding
1979	Oláh Andor [80]	Southeast Hungary	*P. rhoeas*	Flos	Infusion/oral	Cough
			*D. consolida*	Not specified	Topical (bath)	Rheumatism
1980	Ujváry Zoltán [81]	Hungary	*C. cyanus*	Not specified	Decoction/topical (wash)	Ulcer
1983	Petercsák Tivadar [82]	North Hungary	*D. consolida*	Not specified	Infusion (mixed with milk)/oral	Against witchcraft (veterinary: cattle)
1984	Rácz Gábor [83]	Transylvania (Romania)	*C. cyanus*	Flos	Infusion (blend component)/oral	Diuretic therapy
			*P. rhoeas*	Flos (petals)	Infusion (blend component)/oral	Tea corrigent to improve colour
			*D. consolida*	Flos	Infusion (blend component)/oral	Not specified
1985	Péntek János [84]	Transylvania (Romania)	*D. consolida*	Herba	The plant was tied to the horn of the livestock on the opposite side of the sick eye	Cataract (veterinary: cattle)
1985	Kóczián Géza [85]	Transylvania (Romania) and Southwest Hungary	*P. rhoeas*	Flos (petals)	Infusion/oral	Sleep-inducing, pain relief for stomach pain
				Semen	Decoction/oral	Stomach pain, internal purifying therapy, smooth muscle spasm relief
			*D. consolida*	Herba	Infusion/oral	Cough, tuberculosis
					Tincture (in brandy)/oral	Heavy menstruation bleeding
					Fumigation/topical	Sick humans and livestock
1986	Tóth József [86]	West Hungary	*P. rhoeas*	Flos (petals)	Infusion/oral	Tranquilizer, throat rinse
			*C. cyanus*	Flos	Infusion/topical, oral	Eye inflammation, heart palpitations, high blood pressure
					Fumigation	Air disinfection
			*D. consolida*	Flos	Not specified	Cough sedative, vasodilator
1989	Tisovszki Zsuzsanna [87]	Central Hungary	*P. rhoeas*	Flos (petals)	Infusion/oral	Cough
1991	Gelencsér József [88]	Central Hungary	*C. cyanus*, *D. consolida*, *P. rhoeas*	Herba	Decoction/topical (bath)	Evil eyes (children)
1993	Lenkey István [89]	North Hungary	*D. consolida*	Herba	Infusion/oral	Cough, common cold, pneumonia, gastrospasm
2000	Gub Jenő [90]	Transylvania (Romania)	*C. cyanus*	Herba	Infusion/topical (wash)	Wound
2001	Bartha Júlia [91]	East Hungary	*D. consolida*	Not specified	Infused wine/oral	Heavy menstruation, vaginal discharge, venereal diseases, nervousness
2002	Ujváry Zoltán [92]	Upper Hungary (Slovakia)	*D. consolida*	Not specified	Not specified	Eye inflammation
			*P. rhoeas*	Not specified	Not specified	Bleeding (unspecified)
2005	Szabó László Gy. [93]	Hungary	*C. cyanus*	Flos	Not specified Infusion (blend component)	Diuretic, throat rinse Tea corrigent (to improve colour)
			*D. consolida*	Flos	Not specified Infusion (blend component)	Laxative, vasodilator Tea corrigent to improve colour
				Semen	Not specified	Purgative, diuretic, vermifuge
			*P. rhoeas*	Flos (petals)	Not specified	Mild sedative, expectorant
2010	Horváth Katalin [94]	Transcarpathia (Ukraine)	*P. rhoeas*	Not specified	Infusion/oral	Bleeding (unspecified), cough
				Not specified	Infusion, poultice/topical	Eye inflammation
2011	Grynaeus Tamás [95]	Southeast Hungary	*P. rhoeas*	Flos (petals)	Not specified	Common cold, cough
2018	Papp Nóra [96]	Transylvania (Romania)	*C. cyanus*	Flos	Infusion/topical, oral	Eye inflammation, earache, hearing loss

**Table 2:** Records on the uses of arable wildflowers in harvest festivals from the 19th century in the Carpathian Basin.

Relevant Period	Source	Region (Current Country)	Date	Species	Name	Description
19th–20th c.	Kapronyi Teréz [97]	North and West Hungary	End of harvest (mid-July)	Arable wildflowers	Harvest feast	A harvest wreath was made of ears, arable wildflowers, colourful bandannas, and paper ribbons
1850s	Prónay Gábor [98]	Hungary	End of harvest (mid-July)	Arable wildflowers	Harvest feast	A wreath made of ears and arable wildflowers was taken to the landlord in a formal march
1850s	Bozena Nemcová [99]	North Hungary (Slovakia)	End of harvest (mid-July)	Arable wildflowers	Harvest feast	A harvest wreath made of ears and arable wildflowers was given to the landlord by the nicest couple among the harvesters
Mid 19th–late 20th c.	Kapronyi Teréz [97]	Hungary	Start of harvest (late June)	*P. rhoeas*, *C. cyanus*	Binding ceremony	A bunch of ears with a poppy and cornflower was tied to the hand of the landlord amidst good wishes
1870s	Ébner Sándor [100]	Transylvania (Romania)	Evenings during harvest (late June–mid-July)	Arable wildflowers	Harvest feast	Harvesters wore colourful wreaths made of arable wildflowers
1890s	Kovács Bálint [101]	Transylvania (Romania)	End of harvest (mid-July)	*C. cyanus*	Harvest feast	A wreath made of ears and the cornflower was placed on the head of the funniest harvester who carried it to the landlord
1900s	Platthy Adorján [102]	North Hungary	End of harvest (mid-July)	Arable wildflowers	Harvest feast	A harvest wreath made of ears and arable wildflowers was ceremonially given to the landlord
1900s	Illés Péter [103]	West Hungary	End of harvest (mid-July)	Arable wildflowers	Harvest feast	A harvest chariot was decorated with ears and arable wildflowers
Early 20th c.	Manga János [99]	North Hungary	Start of harvest (late June)	Arable wildflowers	Binding ceremony	A bunch of ears with wildflowers was tied to the hand of the landlord who gave money in exchange for drinks
		North Hungary	End of harvest (mid-July)	Arable wildflowers	Harvest tradition	A bunch of ears with arable wildflowers was taken home and hung on a wooden beam and its seeds were used as sowing seeds in the following autumn
		West Hungary	End of harvest (mid-July)	Arable wildflowers	Harvest tradition	A wreath formed from ears and arable wildflowers was laid around the neck of one of the harvesters, then it was taken home and given to hens to increase egg laying
Early 20th c.	Gelencsér József [88]	Central Hungary	End of harvest (mid-July)	*P. rhoeas* and *C. cyanus*	Harvest feast	A wreath made of ears, the poppy, and cornflower was ceremonially given to the landlord
1930s	Faggyas István [104]	North Hungary	Start of harvest (late June)	*C. cyanus, D. consolida*, *Vicia* sp.	Binding ceremony	A bunch of ears with the cornflower, larkspur, and wild vetches was tied to the hand of the land steward by a young girl amidst good wishes
		North Hungary	End of harvest (mid-July)	Arable wildflowers	Harvest feast	A harvest wreath was made of ears, wildflowers, and colourful paper ribbons
1930s	Gyimesiné Gömöri Ilona [105]	North Hungary	End of harvest (mid-July)	*P. rhoeas*, *C. cyanus*	Harvest feast	A wreath made of ears, the poppy, and cornflower was ceremonially given to the landlord
1940s	Illés Péter [103]	West Hungary	End of harvest (mid-July)	*P. rhoeas* and *C. cyanus*	Harvest feast	A harvest wreath was created from ears and arable wildflowers, and the stage was also decorated with the remaining flowers.
Early 1950s	Illés Péter [103]	West Hungary	End of harvest (mid-July)	*P. rhoeas*, *C. cyanus*, *A. githago*	Harvest feast	Two wreaths from ears, the poppy, cornflower, and corncockle were made. One was taken to the grave of the previous landlord and the second was given to the new one

**Table 3:** Records on the uses of arable wildflowers in religious ceremonies from the mid-19th century in the Carpathian Basin.

Relevant Period	Source	Region (Current Country)	Date	Species	Name	Description
Mid 19th c.–today	Sz. Tóth Judit [106]	German (Swabian) communities near Budapest	21 May–25 June	*D. consolida* and other unspecified wild flowers and garden plants	Corpus Christi	Wreaths and bouquets from the plants are placed next to the altar or hung on the wall of the chapel to increase their remedial power
Mid 19th c.–today	Sz. Tóth Judit [107]	German (Swabian) communities near Budapest	21 May–25 June	*C. cyanus*, *Leucanthemum vulgare*, and other unspecified arable wild flowers, *Sambucus* sp., *Robinia* sp., *Sedum* sp., garden plants	Corpus Christi	Flowers are gathered and flower carpets are laid on the route of the procession. Flower wreaths are also made and hung on chapels and tents
Early 20th c.	Horváth Iván [108]	Croatian communities in West Hungary	21 May–25 June	*P. rhoeas* and other unspecified wildflowers	Corpus Christi	Petals of wildflowers, especially those of the poppy, were gathered by children and were thrown on the route of the procession
Early 20th c.	Kalapis Zoltán [109]	Vojvodina (Serbia)	21 May–25 June	*P. rhoeas*, *D. consolida,* and unspecified grasses	Corpus Christi	Freshly mown poppy and larkspur together with grasses were transported from the fields and meadows with carts to decorate the streets
Early 20th c.	Demeter Zsófia [110]	Central Hungary	21 May–25 June	*P. rhoeas*, *C. cyanus*, *Leucanthemum vulgare*	Corpus Christi	Hay, poppy, cornflower, and marguerite were thrown on the route of the procession
Early 20th c.	Nagy Netta [111]	Southeast Hungary	21 May–25 June	*P. rhoeas*, *Rosa* sp.	Corpus Christi	Previously petals of the poppy, later those of the rose, were thrown by young girls during the procession
1930s	Császi Irén [112]	North Hungary	21 May–25 June	*A. githago*, *P. rhoeas*, *C. cyanus*, *L. tuberosus*	Corpus Christi	Petals of wildflowers, mainly those of the poppy, cornflower, corncockle, and tuberous pea were gathered and thrown by young girls during the procession
1930s	Illés Péter [103]	West Hungary	2 July	Arable wildflowers	Visitation of Our Lady	During the service of thanksgiving, the priest blessed the harvest tools that were decorated with ears and arable wildflowers
Early–late 20th c.	Bencsik János [113]	Romanian and Serbian communities in South Hungary	24 May–27 June	*P. rhoeas*, *C. cyanus*, green wheat	Wheat blessing during Orthodox Pentecost	Wreaths were prepared from the corn poppy, cornflower, and green wheat, after which they were placed into wells (to prevent water pollution) or fed to livestock (to prevent diseases)
		Romanian and Serbian communities in South Hungary	24 June	*G. verum*, *P. rhoeas*, *C. cyanus,* other wildflowers and garden plants	Ivana Kupala (John the Batist) day	After the religious ceremony, flowers were gathered by children to make wreaths with magical powers of fortune-telling

## 3. Results and Discussion

Altogether, we found 108 publications discussing the ethnobotanical uses of the three studied arable weed species in the relevant Hungarian literature published between 1578 and 2021. In terms of their medicinal usage, we found 100 records in 43 documents. Among these notes, 42 refer to *P. rhoeas*, 39 to *D. consolida*, and 19 to *C. cyanus*. There is a great variety, both in the therapeutic purposes and mode of application, of each species (Table 1). *P. rhoeas* stands out with its sedative influence, *D. consolida* was primarily applied to stop bleeding, while *C. cyanus* was most frequently used to cure eye inflammation. In relation to food items, *P. rhoeas* was mentioned in four publications, as a famine food, delicacy, or food dye; while *D. consolida* was mentioned in two papers, as a food colouring or a delicacy. Four papers asserted the melliferous potential of *C. cyanus*, and one paper suggested its potential for grazing livestock. Ten articles highlighted the importance of these species as ornamental plants. Twelve papers described the role of these wildflowers in rituals and traditions related to (cereal) harvest, while their role in religious festivities, especially in Corpus Christi, were reported in ten. The cultural significance of these species for children’s games and toys, particularly that of *P. rhoeas*, was presented in 13 studies. We also found 20 studies that discussed the role of the studied species in folk art. These plants are also regularly depicted in the visual arts and literature—from which a few iconic ones will also be discussed to illustrate the symbolic meaning and metaphorical applications of these species and to highlight the deep cultural embeddedness of these charismatic arable weeds.

### 3.1. Medicinal Uses

The first written records between the 16th and 17th centuries concern only *P. rhoeas* and cover a very broad scale of remedial power from insomnia through gum diseases, intestinal pain, St Anthony’s fire (erysipelas), and bleeding (Table 1) [55,56,57]. In the late 18th century, this plant was considered a panacea [62]. At the same time, physicians started to recommend it for respiratory disorders [61] and the plant later became a general cough reliever in Hungarian folk medicine [68,75,79,80,87,94,96]. *P. rhoeas* also used to be listed in many European dispensatories as ‘syrupus rhoeados’, a sweet infusion made of poppy petals that was most notably used as a red colouring in pharmaceutical mixtures [114]. Poppy heads were also widely used as a sedative-hypnotic tool to calm crying babies, who would suck on this “pacifier”, producing sounds such as “peep-peep” while gradually sinking into a deep sleep [52]. According to Rácz [52], this practice was so widespread that these baby sounds can even be related to the etymologic origin of the Hungarian name of the poppy (“pipacs”).

The earliest Hungarian hint of the medicinal application of *D. consolida* is in a set of handwritten margin notes from the very beginning of the 18th century: “*When the blood of your nose starts to run heavily*, *take the herb called larkspur growing among wheat*, *dry it*, *smash it*, *and blow it into the nose with vinegar.*” [58]. Even though this plant was later suggested to treat many different ailments, its most important application was to halt diverse types of bleeding, including wounds and gynaecological complaints, as a topical treatment [65,66,69,71,73,76,84,85]. While most sources do not specify the type of bleeding, in many cases, the context suggests gynaecological problems. The plant was prepared and administered in diverse ways against bleeding. For example, its herb would have been boiled together with bathwater and served as hot as the patient could bear, or it could also be used “secretly”, smuggled under shoe inserts to cure an unspecified bleeding [71,76]. In some cases, the larkspur was also administered internally when its decoction was mixed into red wine [65], brewed together with beer [66], or extracted in a brandy tincture [85]. In some Hungarian regions, fumigation with *D. consolida* was conducted to cure gynaecological bleeding or to prevent premature childbirth, and it was also applied as a component of breast plasters in the case of mastitis [65].

In Hungarian folk medicine, the flowers of *C. cyanus* were used as a poultice for eye inflammation since the 18th century [60,61,63], and this stayed in use as its most common application until the middle of the 20th century [78,86,92,94,96]. Table 1 shows that it was also utilized in diuretic therapies [78,79,83] and we also found a record demonstrating that it was used to mitigate heart palpitations [86].

**Figure 2:**
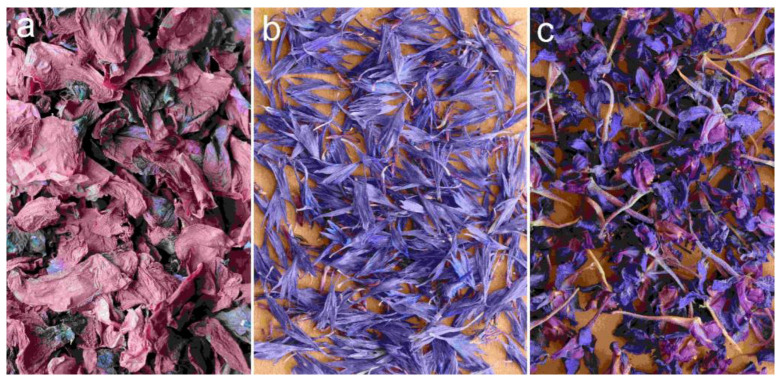
Traditional medicinal products from the three studied species, including (**a**) dried petals of corn poppy (*Rhoeados flos*); (**b**) ray florets of cornflower (*Cyani flos*); and (**c**) tepals of field larkspur (*Calcatrippae flos*) (Photographs by Gyula Pinke).

Related to the collection of these herbs, there is an interesting observation from 1798 in a book by Veszelszki [63]: “*Hard-working fathers and mothers send their children, who are regarded too weak to bear heavy work, to pick poppy flowers in the fields in midsummer, which they sell, or dry in sites where there is no sunlight*.” This suggests that this plant was gathered and processed mainly by children at the turn of the 18–19th century. Later, during the Great War, there was a special ministerial decree to oblige school-teachers to organise their pupils to harvest medicinal plants (particularly the poppy and cornflower) during the summer holiday, which was intended to mitigate the general scarcity of medicaments in the era [115]. According to the yearly Hungarian pharmaceutical bulletins of the era, the poppy, cornflower, and larkspur were among the most popular medicinal plants gathered from the wild, which could be sold at a relatively good price until the 1960s. While the direct pharmaceutical usage of these drugs was gradually declining, they were still important for the beauty industry, mainly as components for face creams [116]. Today, these plants are rarely used for medicinal purposes and the poppy is even regarded as obsolete in modern phytotherapy. However, all these plants are still used in herbal tea blends in small quantities as minor “corrigents” to improve the colour of the infusion (Figure 2) [117].

*P. rhoeas* was also extensively used as a soothing agent for various ailments in Poland [18], Serbia [118], Italy [119,120,121,122,123,124,125,126,127], Greece [128], Spain [129,130,131,132], and Turkey [133,134,135]. The poppy was also used outside Europe, e.g., in Morocco [136], Algeria [137], Tunisia [138], Iraq [139], and Iran [140]. In most cases, the flower, or less frequently its seeds, was prepared for traditional medicines. In Italy, a poppy was also administered to crying babies to induce sleeping but in the form of a decoction [120], while, in Kosovo, an infusion made of poppy seeds was used for a similar purpose [141]. The use of a larkspur as a remedy was also documented in Switzerland: if a woman suffered from discharge, she placed a larkspur in her shoes and kept it there for three days [142]. In Northwest Europe, chirurgeons also applied a larkspur on wounds and broken bones [142]. Wound treatments with *D. consolida* were also performed in Serbia [143] and Catalonia [144], and the plant was also used for diuretic therapies in Romania [145] and Italy [146]. The cornflower was also valued as a disinfectant for eyes and wounds in the Renaissance herbals of Western Europe [142], and it was used as a folk remedy for eye diseases in Belarus [147], the Polish–Lithuanian–Belarusian borderland [148], Kosovo [141], Bosnia and Herzegovina [149,150], Italy [122,151], Spain [144], and Armenia [152]. Furthermore, it was also used in diuretic therapies in Belarus [147], Ukraine [153], and Bulgaria [154], and to relieve heart palpitations in Italy [155]. The plant was also used for blood purification and cleansing the respiratory tract by traditional Polish herbalists [156], as well as in Lithuania [157] and Kosovo [141]. In Ukraine, cornflower tea was once considered as a panacea [158]. All these plants are still used in herbal tea blends in small quantities as minor “corrigents” in some European countries [142,159].

### 3.2. Food Uses

There are only a few records in Hungarian literature mentioning that the studied species were directly consumed as food items. The large buds of *P. rhoeas* were sporadically consumed as a famine food in the 19th century [160], but in some regions they may have also been consumed more regularly [161]. Two cases of accidental poppy poisoning of children were reported in the Austrian–Hungarian borderland in the early 20th century by Barsi [162], who suspected that more undocumented cases of fever and dazed sleepiness among children may have been caused by the consumption of these attractive, large, and apparently delicious buds. According to Barsi [162], poppy consumption may have spread to Western Hungary from neighbouring Styria and Carinthia, where the plant used to be consumed as a vegetable, even as pottage.

**Figure 3:**
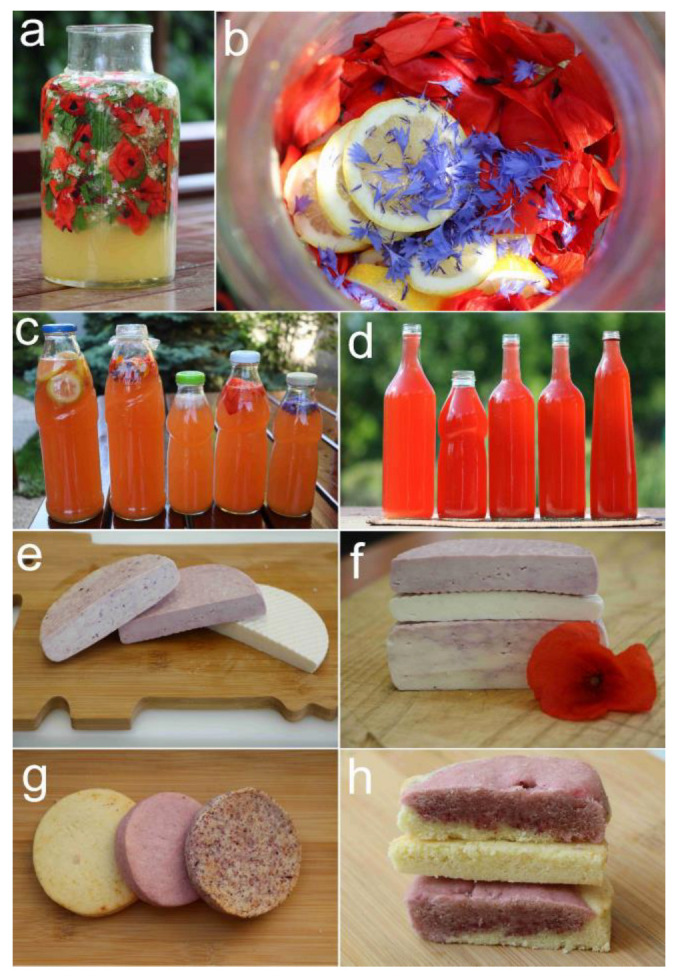
Food applications of the studied species, including poppy petals and cornflower flowers, used to dye and decorate homemade black elder (*Sambucus nigra* L.) syrup (**a**–**d**); handcrafted cheese (**e**,**f**); and cakes (**g**,**h**) coloured using poppy petals and petal extracts (Photographs by Gyula Pinke).

The petals of a poppy were gathered not only for folk remedies and pharmaceutical dye, but they were also used to colour cheese, cakes, and wine [163] (Figure 3). Similarly, the flowers of *D. consolida* were also used as a food dye; mixing the original green dye with alum could turn it blue, and both colours were utilized by confectioners [33]. This species was also picked by children, who sucked out the nectar from the long spurs of its flowers [164].

In many European countries, *P. rhoeas* used to have more significant culinary applications. However, in Italy, young poppy leaves are still eaten raw in mixed salads, or cooked in vegetable soups, omelettes, and pizzas [165,166,167,168,169,170,171,172]. Similar usages have also been reported from Croatia [173,174], Bulgaria [175], Greece [176], Spain [177,178], and Turkey [179,180]. From the latter country, there were even recent intoxication cases related to its consumption [181]. In Italy, poppy seeds were used to flavour bread and cookies [171,182]. The poppy was also used as a component of alcoholic drinks in Croatia and in Catalonia [132,174]. Additionally, poppy petals were used as a food colouring in Croatia [183] and as an ingredient for cosmetics, including lipsticks and cheek make-up in Italy [171,184,185]. The bright red colour of poppy petals was also utilized as a fabric dye in Italy [186]. Polish ethnobotanists reported that the very young shoots of *C. cyanus* were added to non-sour soups, while its flowers were combined with sugar to make wine and beer; moreover, they were also used to dye vinegar [147,173,187]. Cornflower infusion was also used for cosmetic purposes in Italy; it gave a special gloss and blue nuance to grey and white hair [185]. The green pigment from the flowers of *D. consolida* was also used in dying confectionery in many parts of Europe [142].

### 3.3. Fodder and Ethnoveterinary Uses

The arable fields stuffed with the poppy, cornflower, larkspur, and other weed species also provided important bee pastures [188], thus contributing indirectly to a further important food resource (honey). One of the earliest beekeeper books printed in Hungary [189] mentions that honeybees adored *C. cyanus* so much that they visited these flowers in mass quantities even in the period of linden (*Tilia* sp.) blossom. In the first part of the 19th century, this plant was regarded as one of the best melliferous plants, and bees could gather its nectar and pollen for up to six weeks [189], producing large amounts of greenish-yellow, delicious, monofloral honey [190]. Even in the middle of the 20th century, large amounts of *C. cyanus* pollen could still be detected in almost every type of summer floral honeys in Hungary [191]. Although *P. rhoeas* does not produce considerable amounts of nectar, it was also significant for beekeepers due to the large amount of pollen gathered by honeybees [190]. Among the three studied species, *C. cyanus* was also reported to have been used as an occasional livestock feed grazed on by sheep [192]. Occasionally, *P. rhoeas* was also foraged by animals, but it could be severely toxic, causing spasms and intestinal pain, sometimes with lethal consequences [193].

The larkspur was also applied for veterinary purposes. In West Hungary, its decoction with horse chestnut (*Aesculus hippocastanum* L.) was used to cure haematuria in cattle [73]. In North Hungary, infusion from a larkspur and other plants was mixed with foremilk and was given for newborn calves to secure fertility and milk-benefit, as well as to protect against witchcraft [82]. An interesting magical healing process was reported from Transylvania; in the case of a cataract, a larkspur was tied to the horn of the livestock on the opposite side of the sick eye [84].

The poppy was also used for animal feed in Turkey [179], particularly for rabbits and pigs. In Italy, poppy leaves were given to hens to increase egg laying [171], and, today, its seeds are still used as birdseed [120,186]. In Sicily, poppy flowers were once fed to farm animals in large quantities to stun them before slaughter [194].

### 3.4. Ornamental Uses

**Figure 4:**
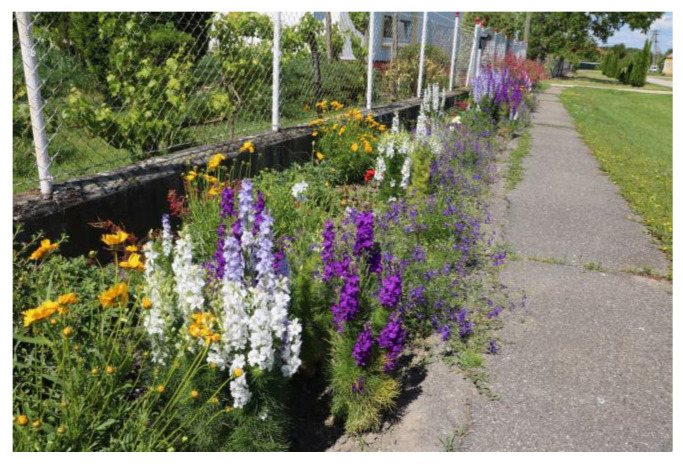
*Delphinium consolida* and *Delphinium ajacis* L. in a street garden (Markotabödöge, NW Hungary, 2020. Photograph by Gyula Pinke).

The first historical record of *C. cyanus* and *D. consolida* being cultivated in Hungary as ornamental garden plants comes from the 17th century [195], but these plants could have actually been grown for ornamental purposes since the 11–12th centuries [196]. *P. rhoeas* was also planted in gardens in the Renaissance era [70] and it was still cultivated sporadically in the 19th century [163]. In the late 20th century, when the arable flora suffered a significant decline [197], the ethnographer, Béla Gunda, observed in many villages that people were striving to “save” the disappearing cornflower: “*Village women gather the cornflower seeds which they sow in their small gardens in the autumn or spring*, *so that this flower*, *a folk favourite*, *could continue to thrive there*” [198]. Today, the seeds of all three species studied in this paper are commercially available in Hungary [199]. Nevertheless, they are relatively rare in gardens, where they have been replaced by more fashionable horticultural cultivars developed from their close relatives (Figure 4). They also used to be popular as cut flowers, providing a small temporary income source to impoverished women [73]. Monofloral and mixed bouquets of these wildflowers were regularly sold in flower markets as recently as the 1950s [200]. In Romania (Transylvania), they are still gathered for decoration in vases [96].

These colourful flowers have also been popular ornamentals in many other parts of Europe. Both *P. rhoeas* and *C. cyanus* have been cultivated since the 16th century throughout Central Europe as garden plants [201,202], which may partly be motivated by a high demand for wreaths [201]. The cornflower was a beloved species in the home gardens of Austria, where it seems to have maintained its high popularity until relatively recently [203]. The diverse horticultural varieties of *D. consolida* have also been common in many gardens of Central Europe [204]. *P. rhoeas* and *C. cyanus* have also been used to embellish bouquets in some regions of Spain [205], and bouquets and wreaths from *C. cyanus* are still sold in open-air markets in Poland [206].

### 3.5. Cutural Uses

#### 3.5.1. Harvest Festivals

**Figure 5:**
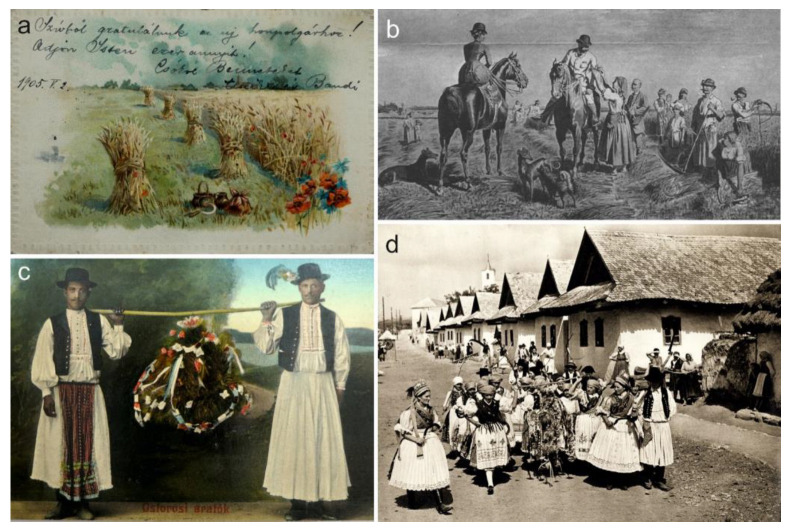
The studied species in historical artworks: (**a**) cereal sheaves with corn poppy and cornflower (postcard with good wishes for a newborn baby from May 1905, collection of Gyula Pinke); (**b**) ceremonial binding of the landlord (drawing by Mihály Szobonya, source: Vasárnapi Újság, 1888, 35 (28): 457; (**c**) harvesters with a harvest wreath (postcard, Ostoros, N Hungary, 1910–1920, © Zempléni Múzeum); (**d**) harvest festival (Kazár, N Hungary, 1940. Photograph by Géza Buzinka, courtesy of Fortepan).

Gál [207] suggests in a short story that wildflowers were important emotional factors in setting the atmosphere of cereal harvests of the past, adding a little colour to the otherwise long and laborious days: “*The harvest goes on like a song*, *it has rhythm and melody. (…) The little flames of the poppies*, *the blue glitter of cornflowers and larkspurs. Every colour*, *every rhythm*, *every beauty all together*” (Figure 5a). These flowers were closely associated with harvest rituals and celebrations, which usually took place both at the beginning and end of the harvest period (Table 2). This period was typically opened with the ceremonial “binding” of the landlord (or steward) during their first visit to the harvesters when the landlord was symbolically tied up with a rope made of the first ears that were decorated with arable wildflowers [99,104] (Figure 5b). The first ears were often regarded to have magical powers, carrying God’s blessings, which would ensure an abundant yield for the next year [97]. The first available report of wreaths made by the harvesters to celebrate the end of the harvest originates from 1806 [208]; however, this record still lacks any mention of wildflowers. Though, by the middle of the 19th century, these plants reportedly became key components of the wreaths [98,99].

According to a note from 1870, harvest workers spent their evenings together singing and wearing colourful wreaths made of various wildflowers during the harvest period [100]. This idyllic image of bucolic harvest celebrations became prominent in many subsequent documents in the late 19th and early 20th century. Nevertheless, it is good to keep in mind that, during this period, the Hungarian government started to recognize the untapped potential of rural traditions in building a national image, and harvest festivals became an important component in this new “country marketing”. During this period, various forms of guidelines and recommendations were issued by various government agencies, sometimes even prescribing the components of harvest wreaths. These recommendations were then mixed with pre-existing traditions at the local level, which makes it difficult to separate the genuine traditions from the new ‘top-down’ trends [209]. The recommendations were outlined in the following protocol: when the toughest part of the work, the reaping, was finished, a large wreath was made from the thickest ears, intertwined with poppy, cornflower, larkspur, corncockle (*Agrostemma githago* L.), and vetch (*Vicia* sp.) flowers, as well as fancy ribbons (Figure 5c). Then, the harvesters carried it through the farm in a solemn march, singing aloud (Figure 5d). The wreath was delivered and handed over to the landlord with a nice speech and, in exchange, he thanked the workers for all their hard effort. Subsequently, the harvest ball could be started with live music, traditional costumes, and a lot of dancing and shouting [88,97,99,101,103,104,105].

In some regions, a wreath or a bunch of ears with all these wildflowers was taken to the homes of the harvesters where it was hung on a wooden beam. These hanging decorations were then left in place for a long time, the maturing seeds of the drying wildflowers were saved and added to the sowing seeds to be used in the following autumn or given to hens to increase egg laying [99].

These flowers used to have similar roles in Czech, Romanian, and Russian harvest celebrations [99,210].

#### 3.5.2. Religious Ceremonies and Rituals

**Figure 6:**
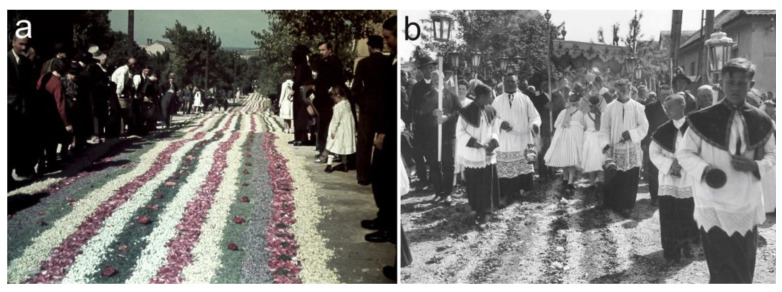
Flower carpet for the procession of Corpus Christi in Budaörs, Hungary (**a**) 1940; by unknown photographer; (**b**) 1943; photograph by Carl Lutz; donated by Archiv für Zeitgeschichte ETH Zürich/Agnes Hirschi. Courtesy of Fortepan).

The three studied wildflowers were also important props in several religious celebrations that took place during the summer months (Table 3). The most significant liturgical event related to arable wildflowers was the feast of Corpus Christi, which is celebrated two months after Easter in the Catholic calendar, typically in late May or early June. The event was celebrated with spectacular processions in several regions of Hungary. To prepare for this feast, children were sent out to the countryside to gather flower petals, especially those of the poppy [108]. Then, during the procession, the priest carrying the Holy Communion was followed by a group of young girls dressed in white holding the petals in small baskets and tossing them around in such an abundance that the ground was often fully covered in a floral carpet (Figure 6a,b). According to the reports of Horváth [108] and Demeter [110], the poppy was preferred because it produced the most spectacular floral carpet, but the flowers of the cornflower, corncockle, tuberous pea (*Lathyrus tuberosus* L.), and marguerite (*Leucanthemum vulgare* Lam.) were also used. Later, the petals of roses (*Rosa* sp.) and peonies (*Paeonia* sp.) became more frequent, and bouquets of garden flowers were also integrated into this celebration. The flowers from Corpus Christi, blessed with the Holy Communion, used to be one of the most respected paraliturgical items for Catholic Hungarians [112]. After the ceremony, these flowers were taken home by the churchgoers to save their houses from lightning strikes or to treat sick children and animals (e.g., via fumigation or infusion baths) [107,108,109,110,111].

In communities that follow Eastern Christian traditions (Greek Catholic, Orthodox), the most important feast involving wildflowers was the birth of John the Baptist (Ivana Kupala). In the Greek-Catholic villages of Northern Hungary, this feast was celebrated with bunches of flowers from the fields, meadows, and gardens that typically included large amounts of the larkspur and cornflower. These flowers were then taken to the church and blessed by the priest at the end of the liturgy (Table 3), which endowed them with magical properties, and they were used for making decoctions, and vapour or fume treatments to heal children and livestock [211]. In the settlements of Southern Hungary, populated by ethnic Romanians and Serbians of the Orthodox faith, John the Baptist (Ivana Kupala) used to be celebrated on 24 June (Table 3). For this event, children and elderly women went to the fields and meadows to pick flowers, which were then bundled into large bunches and wreaths. The mainstay of these wreaths was yellow bedstraw (*Galium verum* L.) but the poppy and cornflower were also common components. These wreaths were flung up onto the thatched roofs and, if they fell off, it was considered to be a bad omen (e.g., prophesising death), while if they remained on the roof, it was believed to be a good sign (e.g., the young girl of the family would get married soon) [113].

The celebration of the Orthodox Pentecost, which usually followed its Catholic counterpart by several weeks, also involves some traditions related to arable wildflowers. In the Romanian and Serbian communities discussed above, Pentecost involved a traditional wheat blessing ceremony where wildflower wreaths were prepared and blessed. These wreaths were later placed into the wells (to prevent water pollution) or were fed to livestock (to prevent diseases) [113].

According to Luczaj [212], floral decorations and a petal toss were featured in Corpus Christi processions in many European countries until the 19th century. Poland seems to be the last refuge for the once widespread tradition of blessing floral wreaths for Corpus Christi in which the most important flowers used are roses; however, the cornflower and poppy can be found in them as well [212]. Cornflower bouquets were also used in Orthodox Pentecostal rituals in western Ukraine. In this region, the seedpods of *P. rhoeas* were used for decoration in other religious festivities (Easter, Feast of the Transfiguration) [213]. In certain regions of Spain, both *P. rhoeas* and *C. cyanus* were also the subject of magical and religious beliefs and practices [205].

#### 3.5.3. Children’s Culture

**Figure 7:**
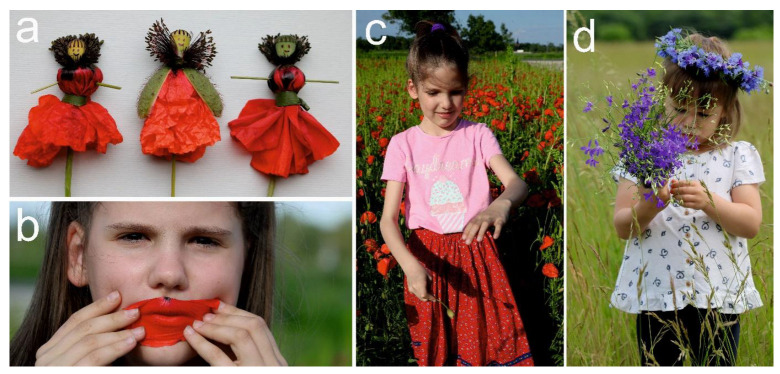
Animated scenes with old-fashioned children’s toys: (**a**) puppets made from poppy flower; (**b**) snapping poppy petals on the lips; (**c**) snapping a poppy bud on the back of the hand (Mosonmagyaróvár, NW Hungary, 2019); (**d**) a cornflower wreath and a bunch of larkspur (Halászi, NW Hungary, 2022) (Photographs by Gyula Pinke).

The relatively large and brightly coloured flowers of the poppy captivated the attention and imagination of children as well. Particularly in smallholder families, where the grandparents, parents, and larger children used to make various types of “poppy puppets” throughout the Carpathian Basin [164,214,215,216,217,218,219]. According to Ortutay [220], the starting point of making a poppy puppet was a poppy bud; first, the sepals were removed, then, the petals were folded down and tied with a blade of grass. This resulted in something that looked like a doll in a red robe (the petals), whose head was the ovary, and the stamens formed a collar. With a few finishing touches, these dolls could be turned into various figures, e.g., an elegant lady, a devil, or the baby of a larger rag doll, which could be used creatively while playing, e.g., as the participants of a wedding or a funeral. In a wedding game, a poppy with white petals was typically used to make the bride, while the red ones were used for the groom and the other guests. In a burial scene, a white poppy was selected for the decedent and the priest was red [164,214,215,216,217,218,219] (Figure 7a). Sometimes, these dolls were dressed up with further accessories, e.g., with a necklace woven of larkspur flowers [164,214].

Moreover, before unfolding a poppy bud, a colour-guessing game accompanied with the nursery rhyme of “*Is it beer, is it wine, is it brandy, or is it a pink ribbon?*” was often played by children in the Carpathian Basin. A poppy with rusty-reddish petals represented “beer”, red was “wine”, white was “brandy”, and a pink poppy stood for the “ribbon”. If the answer was right, the respondent received a treat (e.g., they could eat the petals or received a flower from the questioner), otherwise they had to pay (e.g., with another poppy flower) [161,217,221]. In other popular children’s games, poppy petals were snapped with the lips [222] (Figure 7b) or poppy buds were hit on the back of the hand [70] (Figure 7c). Thus, similar to the German names (e.g., “Klatschmohn”, “Klapperrose”, and “Klatschrose”), the Hungarian name of this plant (“pipacs” and its archaic forms “pippancs” and “papics”) could also be of onomatopoeic origin (closely related to the words “pattint” [snap] and “pacskol” [slap]) [52]. These words probably came into existence before the first half of the 16th century independently of other languages [223], which suggests a long-standing cultural relationship to this plant by the Hungarian ethnic populations living in the Carpathian Basin. The poppy was also frequently represented as a character in tales and poems for kids, often in a protagonist role as the “poppy king”, “poppy lady”, or a “little poppy”. These fabulous heroes usually had a red face, wore red clothes, and made bright, flirtatious appearances [36].

The cornflower was also used to make wreaths by young Hungarian peasant girls [63,84,215] and the decorations made of larkspur flowers were also highly appreciated in wedding games [224] (Figure 7d). On the day of Pentecost, the cornflower was used as a gift of love in some Hungarian regions where lads gave small bunches of the cornflower, sometimes accompanied with other wildflowers, to the girls they liked most [225].

Poppy dolls were also popular among children in Germany and Central Italy [171,204]. Italian children also used to play a colour-guessing game with the still closed flower buds, saying “*frate, monaca o cappuccino?*” (monk, nun, or capuchin?) [171]. In Italy, poppy ovaries and seed pods were also played with as “stamps” that left a nice mark on the skin [171,182]. One of its German folk names (“Tintenblume”) indicates that a red ink could also be made from the petals by the children [204]. Folkloric records suggest a widespread use of the poppy in children’s games within [226] and beyond Europe [227]. The cornflower was also listed in one of the earliest ethnobotanical inventories as a plant used in children’s games in Germany and Upper Austria [228].

#### 3.5.4. Visual Arts

**Figure 8:**
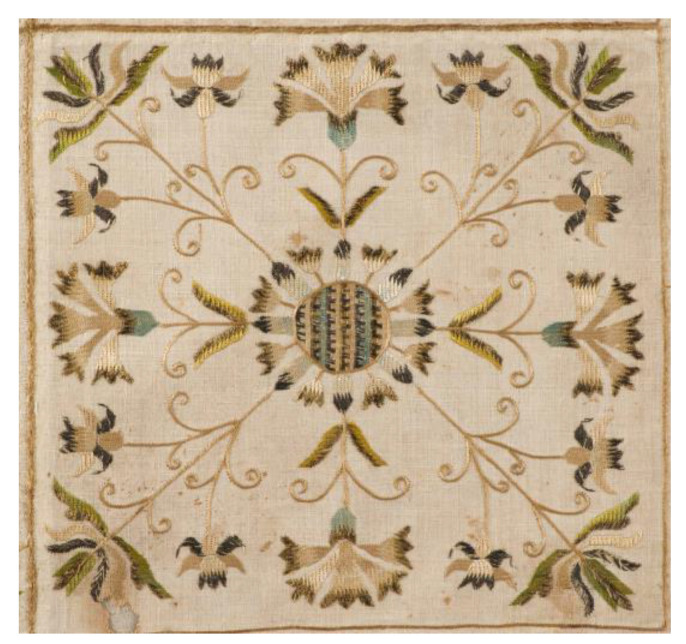
Fragment of a panelled cover cloth (late 17th century, Calvinist church of Marosvécs [Brâncovenești], Transylvania, Romania). Cornflower is located in the centre of the motif from which carnations and tulips emerge. The central cornflower is a favourite stylistic element of Transylvanian embroideries (Museum of Applied Arts, Budapest. Photograph by Áment Gellért. © Iparművészeti Múzeum).

Together with the rose, carnation (*Dianthus* sp.), and later tulip (*Tulipa* sp.), the cornflower has been one of the oldest and most archaic floral motifs in Hungarian folk art [229,230]. The cornflower used to be particularly popular as a Christian motif, appearing regularly in diverse religious contexts, e.g., on painted church ceilings [229] (dated 16th c.), [230] (dated 18th c.); embroidered church tablecloths (Figure 8), [231,232] (dated 17th c.), [233] (dated 1898); altar cloths [234] (dated 17th c.); and vestments [235] (dated 1792), [236] (dated 20th c.). Furthermore, from the 19th century, the cornflower has gradually infiltrated the decoration of household items, including sheets [231] (dated 19th c.); bonnets [237] (dated 19–20th c.); chests [238] (dated 1853); ceramic pots [239] (dated 1926–1929); and horn carvings [240] (dated 19–20th c.). From the 17th century, the motifs of the poppy also emerged, first on pewters [241] (dated 17th c.), but later it became a favourite element on diverse folk embroideries [242] ranging from liturgical tablecloths [243] (dated 1897), [244] (dated 1915) to evening dresses [245] (dated 20th c.). In some regions of Hungary, larkspur flowers also became popular floral motifs on embroideries as well as wall paintings [246].

**Figure 9:**
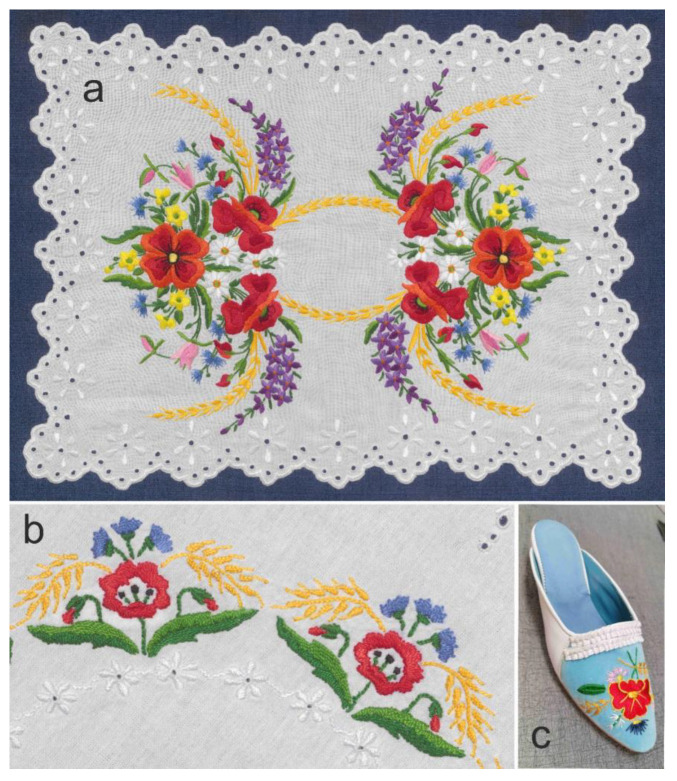
Contemporary folk embroideries with wheat ears and arable wildflowers motifs: (**a**,**b**) textiles from Vojvodina (N Serbia) with motifs from Ada (**a**) and Torontálvásárhely [Debeljača] (**b**) (both manufactured by Veronika Serfőző, photographs by Ágnes Nagy Abonyi. Courtesy of Rozetta Kézműves Társaság, Zenta); (**c**) Slippers from Szeged (S Hungary, courtesy of Sallay Szegedi Papucs).

While these wildflowers do not belong to the most common floral motifs of Hungarian folk art, they are present in several traditional ornamental styles. Cornflower, poppy, and larkspur motifs also appear among the famous patterns of Kalotaszeg (Țara Călatei) in West Transylvania, Romania, probably originating from the 18th century [247]. These wildflowers are also prominent motifs in the embroidery techniques from Torontálvásárhely (Debeljača) (Figure 9a) and Ada (Figure 9b), both developed during the 20th century in Vojvodina, Serbia [248,249,250]. Despite the fact that other, older folk-art styles used strongly stylised floral patterns and were largely detached from any concrete species [251], these new styles applied relatively easily recognizable naturalistic and recognizable figurative floral motifs. In the case of Ada, these motifs include the corncockle, marguerite, and buttercup (*Ranunculus* sp.), in addition to the three studied species [248]. Furthermore, some of these appear on traditional hand-embroidered slippers in nearby Szeged (Hungary, Figure 9c) [252,253]. Traces of these folk-art styles were adopted by more recent “souvenir folk art” designed and mass produced by business ventures trying to meet the demand of tourists [254].

**Figure 10:**
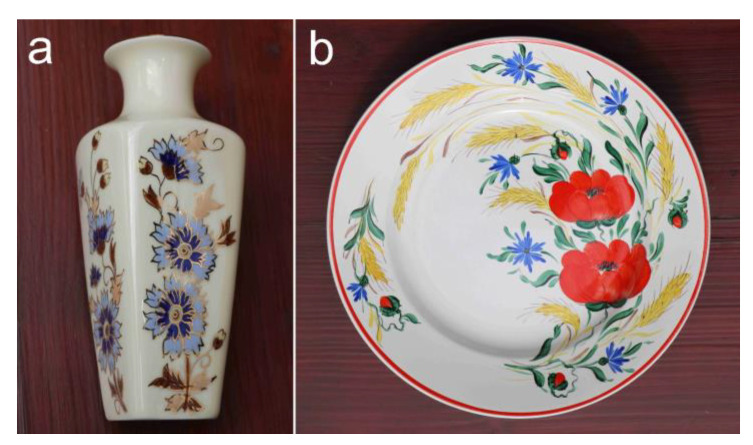
(**a**) Porcelain vase with cornflower motifs (Zsolnay factory); (**b**) Hand-painted wall plate ceramics (Photographs by Gyula Pinke).

**Figure 11:**
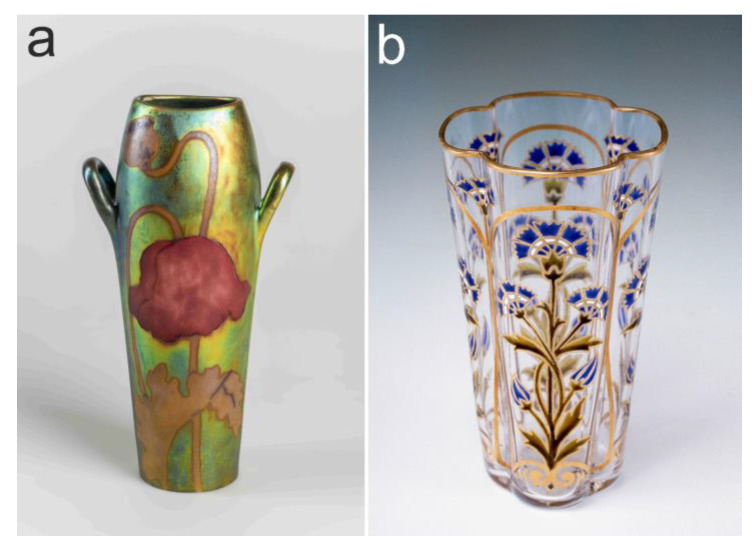
Ceramics from the Hungarian Art Nouveau: (**a**) Vase with red poppies (ca. 1900, Zsolnay factory, Museum of Applied Arts, Budapest. Photograph by Jonatán Urbán and Dávid Kovács. © Iparművészeti Múzeum); (**b**) Cup with cornflowers (1896, Henrik Giergl company, Museum of Applied Arts, Budapest. Photograph by Ágnes Kolozs. © Iparművészeti Múzeum).

**Figure 12:**
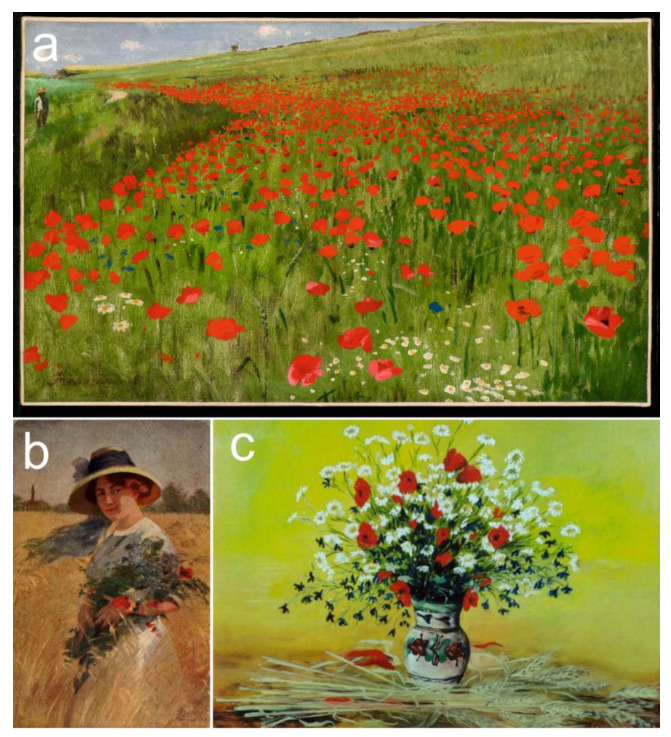
Hungarian paintings featuring arable wildflowers; (**a**) Meadow with poppies (Pál Szinyei Merse, 1896; Museum of Fine Arts, Budapest; © Szépművészeti Múzeum 2022); (**b**) Summer (The reproduction cards of the ‘Publisher Könyves Kálmán’. Reproduced after: A hatted lady with a bouquet of arable wildflowers, Fülöp Szenes, 1913. Courtesy of Bedő Papírmúzeum); (**c**) Bouquet of arable wildflowers (Gizella Czeglédi, 1999. Courtesy of Gizella Czeglédi).

The cornflower is also one of the most popular motifs on a popular high-end product line of the Hungarian porcelain manufacture ‘Zsolnay pottery’ (Figure 10a) [255]. Furthermore, the three wildflowers examined are also often featured on other hand-painted ceramics (Figure 10b). The cornflower and poppy were popular elements of the Hungarian Art Nouveau at the turn of the 20th century (Figure 11). In Hungary, Pál Szinyei Merse is the most eminent painter in terms of depicting emblematic landscapes with vibrant poppies at the end of the 19th century [35]. He created those pictures on his provincial estate, which became masterpieces of Hungarian naturalism (Figure 12a). He was characterized by the art historian Antal Hekler [256] as, “*… a warm-hearted interpreter of the Hungarian reality aflame with poppies*”. The studied three wildflowers were also frequently illustrated in genre paintings (Figure 12b) and still life pictures (Figure 12c).

The three studied wildflowers have also been popular decorative elements in folk art in several other European countries over the last few centuries [18,257,258]. According to Polish-Ukrainian beliefs, the cornflower was one of the favourite flowers of water-nymphs [259]. The cornflower was also often depicted in paintings during the Middle Ages and the Renaissance period, frequently seen in Christian frescos as a symbol of Mary or Christ [17,142]. In modern art, the French impressionist, Claude Monet, garnered worldwide fame with vibrant poppies in his paintings [260].

#### 3.5.5. Literary Works

Sándor Petőfi, the most famous Hungarian patriotic poet of the 19th century, wrote: “*The fields are filled with flowers, you will find/Poppies grow in gay profusion/All genera, every kind*” (“*Szántóföld szépen virít,/Termi bőven a pipacsnak/Mindenféle nemeit*”). In this satirical poem, published in 1847, the thriving poppies refer to the negligence of lazy Hungarian landlords of the era. Another famous epic of Petőfi, titled “*John the Valiant*” (“*János vitéz*”, 1844), was transformed into an opera by the composer Pongrác Kacsóh in 1904. In this opera, the poppy emerges as a symbol of homesickness and patriotism. At the climax of this piece, the sound of a flute touches John’s heart, who says: “*Back home, poppies and larkspurs have started to bloom, by the time I get home it will be time for harvest…*”.

In the first half of the 20th century, the poppy, sometimes also the cornflower and larkspur, appears as metaphorical illustrations of the Hungarian homeland and folk spirit [35]. “*Hungary, my beautiful mother country with poppy flowers and wheat ears*”—passionate exclamations, similar to this one written by Mici Gruber (1928), were quite common in public magazines of the era. In the early 20th century, this flaming red flower could also symbolize the increasingly popular revolutionary political movements. “*The rich fields are set ablaze in poppies/by the fiery wonder of the Hungarian summer*” (“*Pipacsot éget a kövér határra/A lángoló magyar nyár tűzvarázsa*”)—this is the iconic beginning of one of the most famous poems of Gyula Juhász in 1918, where the poppies in the glowing landscape became an impressionist personification of the growing societal tensions, possibly heading towards an imminent revolution [261].

**Figure 13:**
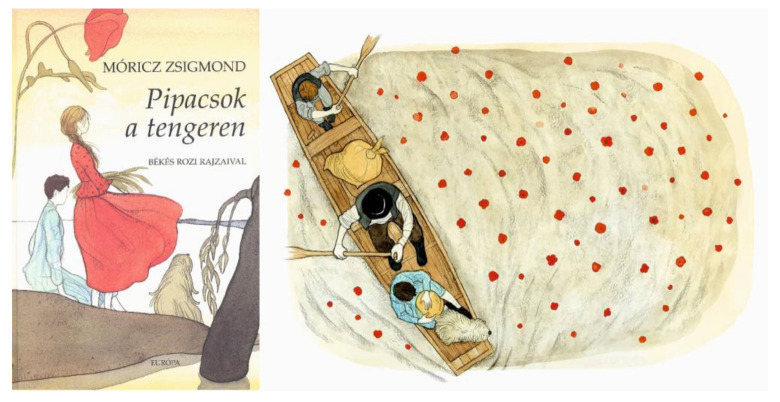
Drawings by Rozi Békés for the illustration of Zsigmond Móricz’s novel “*Poppies on the sea*” (1908), where a major flood on the Tisza river causes the red flowers to float (Courtesy of Rozi Békés).

One of the novels by Zsigmond Móricz, a famous writer of the early 20th century, titled “*Poppies on the sea*” (“*Pipacsok a tengeren*”) (1908), takes place on the Great Hungarian plain, which was flooded by the Tisza river, and the flowers were floating on the surface (Figure 13). In this story, a little boy, who is the writer himself, falls in love with a little peasant girl wearing a red skirt, and secretly calls her “*my little blood red poppy flower*” (“*Kis vérszín pipacsvirágom*”). This suggests that the plant can also be associated with emotional infatuation. Poems by Károly Szász (1930) make a similar association, connecting the poppy to burning love: “*Poppies were burning in the grass/blazing like your kisses/red like my blood/I stuck a poppy in your hair*” (“*Pipacsok égtek lángolva a fűben./Tüzesek, mint a csókod,/Pirosak, mint a vérem./Egy pipacsot én a hajadba tűztem*”). This passion is further intensified by Lajos Nechanszky (1932): “*Your lips, the trembling blood red poppies/(…) are whispering their glowing embers at me” (“Imbolygó, vérszínű pipacs a szád/(…) és rámsusogja forró parazsát.*”).

Nevertheless, due to the short life and quick fall of its petals, this plant was also considered as a metaphor of impermanence, and the resulting sorrow and lovesickness. As Mihály Tompa illustrates (1853): “*Oh, its ornaments are so perishable/they blow in the morning and fall before the evening!*” (“*Ah, de dísze oly múlandó./Reggel nyílik, estig elhull*!”). In his poetry book titled “*When you were poppies*”, László Király (1982) makes a dramatic observation: “*Youth is gone in a flash of red poppies*” (“*Tovatűnt az ifjúság pipacsszínű lobbanása*”) [262].

The cornflower was often used with nostalgic intent, to invoke the intimate atmosphere of harvests from days gone by. For example, Ferenc Mátyás (1952) wrote: “*Cornflowers in the girls’ hair/flames in their eyes and hearts/their songs tear sorrow apart/as they are binding wheat into sheaves*.” (“*Búzavirág a lányok hajában,/szemükben is, szívükben is láng van./Száll a daluk a bút összetépve,/úgy kötözik a búzát kévébe.*”). According to an old legend [37], possibly originating from Western Europe [142], God made this flower blue, so that bent-over peasants could still admire the colour of Heaven during their tough work in the fields. This legend can also be traced back to a metaphor by Ferenc Mátyás (1952): “*As if the sky broke into pieces/and it would shine down here*” (“*Mintha az ég darabokra törne,/s csupakéken itt lenn tündökölne”*). Sometimes, the flowers came with a stronger religious meaning, as illustrated by József Erdélyi (1935): “*Your blue colour, like the clean, almighty sky/is an ethereal virgin faraway!*” (“*Kék színed, mint a tiszta, magas ég,/a földöntúli, szűzi messzeség!*”). István Toronyi (1932) also refers to the divine origin of this plant: “*Holy water dropped to your nice blue clothes/when you became in holy baptism/cleansed from sin: the flower of God.*” (“*Szenteltvíz hullott szép kék ruhádra/S akkor lettél te szent keresztségben/Bűntől tisztulva: Isten virága*”). Accordingly, the cornflower used to be a universal symbol of innocence, virginity, perseverance, and faithfulness [263,264,265]. At the same time, this plant was often used as a metaphor of gentle and tender love, as Jenő Dsida (1930) expresses: “*I silently sigh the blue love of cornflowers towards you*” (“*Csöndesen feléd sóhajtom a búzavirágok kék szerelmét*”).

The deep relationship between the larkspur and farmers is reflected in the works of several poets from a peasant descent [38]. As György Dénes (1961) illustrates: “*I am walking on peasant-fields again/… larkspurs are gently guiding my path*” (“*Paraszt-mezőkön járok újra/(…) szarkaláb hajlik/szelíden útamra*”). This plant could also encapsulate nostalgic memories, suggesting that this flower could be an important element in making an imprint on youth, as it is in the case made by Imre Oravecz (1997): “*You also used to be a child/holding a quail chick in your hands/walking barefoot on soft grounds/picking larkspur at sheaves binding*” (“*Voltál gyermek,/tartottál kezedben fürjfiókát,/lépkedtél mezítláb a föld puha hátán, /szedtél szarkalábat marokveréskor*)”. Similarly, Dániel Hatvani (1965) also mentions this plant evoking an old romance: “*Only the stacked sheaves of wheat might/keep the larkspur-scented memories of first loves/… threshing machines murmuring in the dust/glittering bodies of girls in the evening sun*” (“*Talán csak búzaasztagok őrzik/az első szerelmek szarkaláb-illatát/(…) mormoló cséplőgépek a por halmazában/izzadó leánytesteken csordult szét a nap*”).

#### 3.5.6. Societal Symbols

In the previous sections, we have reviewed cultural and artistic applications of the three studied species among the Hungarian-speaking communities in the Carpathian Basin. All these cultural applications, whether they are traditional ceremonies or artistic motifs, are based on the symbolic meanings that these species convey. In this section, we summarize these symbolic messages (Table 4), connecting them to further social movements and phenomena.

**Figure 14:**
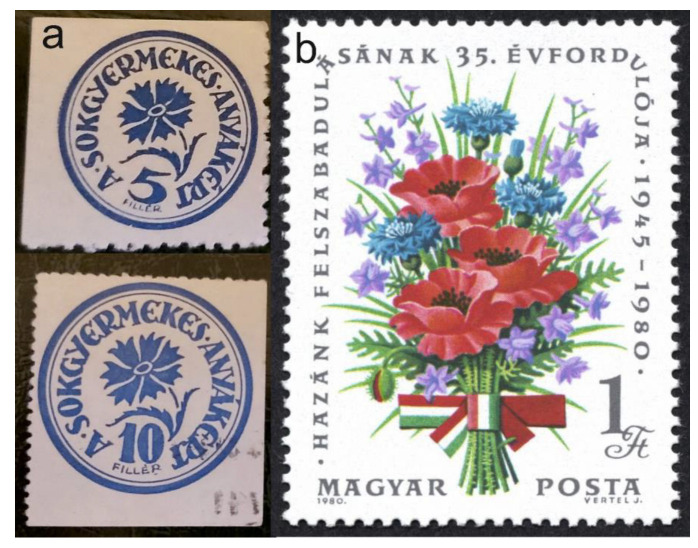
Stamps with cornflower motifs: (**a**) “*For the mothers with many children*”, a series of charity stamps issued by the “*cornflower-action*” movement (1929–1939) (Courtesy of Bedő Papírmúzeum); (**b**) a Hungarian postal stamp with arable wildflowers (1980) (Designer József Vertel, Courtesy of Magyar Posta).

As we discussed above, all three species have often symbolised a bucolic nostalgia and yearning toward a simple rustic life, the idyllic reminiscence of a lost homeland or youth. Nevertheless, in the early 20th century, the three studied arable weed species also became patriotic symbols of the consolidating Hungarian state and the Hungarians in it that were seeking their identity in the dualistic Austrian-Hungarian monarchy. At this time, the poppy and cornflower were seen as the most important components of wreaths and bouquets used in summer burial ceremonies to decorate a coffin, hearse, and even streets where the funeral procession of a prominent public figure passed by. These flowers symbolised the connection that tied the decedent to the Hungarians [35,37]. This period coincides with a Europe-wide renewal of symbolic systems, with a proliferation of new national symbols all over Europe [266]. Nevertheless, the poppy, cornflower, and larkspur remained hidden but popular national symbols in Hungary during the era of socialism (Figure 14b) and afterward [267].

After the First World War, the poppy became a particularly important symbol representing the blood shed by the soldiers, but also a hope for regeneration and renewal. This symbol probably has multiple roots: poppies were reportedly abundant in the disturbed landscapes of the battlefields [268,269]. Their colour allowed for an easy association with the blood shed and the short-lived flowers also provided a natural allegory of transience and fragility, which has also been documented in this study (Table 4). The poppy, as a metaphor, was also sporadically used in Hungarian war coverage from the Eastern front, as poppies growing on soldiers’ graves resembled the blood drops of the fallen soldiers [35]. Moreover, the unusually high abundance of the poppy in Hungarian arable fields in 1916 was explained in a contemporary article as the “*blood of the Earth overflowing in sorrow (…), as its sons are falling in the Eastern and Italian fronts (…), far away from their motherland”* [35]. Accordingly, the poppy became an important symbol of the huge and heroic, possibly pointless losses, and this meaning is preserved in several national symbols of remembrance still actively used today, including the “*Flanders poppy*”, or the emblem of the Royal British Legion [268,270].

The association of ephemerality with the poppy might go back to very ancient roots—as suggested by Beuchert [201], who identified the poppy wreaths found in the grave of a young Egyptian princess as an indication of “fragile and evanescent existence”. In Ukrainian Carpathian folklore, this short-lived flower is also associated with transience, briefness of youth, and unfortunate love [271]. Moreover, the poppy was also seen as a symbol of pride (due to its impressive display) and of sleep (based on its popular medicinal use) [142,272].

As discussed in the previous sections, the symbolic meanings of the cornflower are much more connected to primary human and societal values including innocence, perseverance, faithfulness, and loyalty (Table 4). Accordingly, it is not surprising that the sky-coloured cornflower was so often used as a symbol in religious contexts. The colour of the cornflower is often explained to have a celestial origin: for example, according to a British legend, the sky sent bits of itself down to the fields, thus creating cornflowers [142]. The cornflower has also been used as a symbol of charitable movements, with a noble societal purpose. For example, the “cornflower-action” was a Hungarian charitable movement between 1929 and 1939, aimed at subsidizing Hungarian mothers with many children by selling paper cornflowers and stamps with a cornflower illustration (Figure 14a) [37,273,274]. Similarly, in Germany, where this plant has a remarkable cultural appreciation [275], artificial cornflowers were also prepared and sold to support old veterans of the Franco-Prussian War in 1870–1871 [37]. As the cornflower also thrived in the battlefield landscapes, this flower was also used as a symbol of remembrance [269]. After World War I, pin badges with a poppy (e.g., in Britain) and a cornflower (e.g., in France) were made (mainly) by disabled soldiers for the purpose of supporting war orphans and veterans [36,37,142]. These charismatic wildflowers can be spotted even in present-day national symbols: for example, the colours of the French tricolour flag are often linked to the poppy and cornflower [142], while the blue of the Estonian flag is also often linked to the cornflower, which is also one of the main national symbols of this relatively young state [276].

Similar to the poppy, the cornflower was also widely used as a symbol of love. Nevertheless, aligned with its general symbolic meanings (perseverance, faithfulness, purity) [201], the cornflower symbolized a slower, more permanent, reliable, and tender emotion. Not surprisingly, around the turn of the 20^th^ century, “*Cornflower*” was a common code word in personal ads in Hungarian newspapers, and it was also one of the most frequently used nicknames in salutations and signatures of secret love messages (“*To my cornflower*”, “*Your cornflower*”, etc.) [37]. As Erdélyi [277] pointed out, the cornflower (as well as the flax—*Linum usitatissimum* L.; and the blackthorn—*Prunus spinosa* L.) appears conspicuously frequently in Hungarian folk poetry describing an ideal (or desired) eye colour. Independent of hair colour, blue eyes were always considered to be signs of tenderness, faithfulness, and serenity in girls. These subconscious idealistic images may explain why folk art so often depicts girls with blue eyes [277]. Cornflowers were also used in various traditions of foretelling love in Western Europe [142].

## 4. Conclusions

Our review explores the long-established and deep cultural embeddedness of the studied three iconic arable weed species, *P. rhoeas*, *C. cyanus*, and *D. consolida* in the Carpathian Basin. Ethnobotanical records and historical artefacts suggest that these species were used on a broad scale among the Hungarian populations of the Carpathian Basin from (at least) the 16–17th centuries until modern times as medicinal, food, ornamental, and cultural resources. These species were emotionally linked to the peasant lifestyle in many ways, providing inspiring symbols for Hungarian literature and visual arts, as well as broader society. Many aspects of these cultural connections peaked in the early 20th century. The drop in the number of records from the late 20th and the 21st century is probably attributable to a combination of the declining diversity of arable weeds and the disappearing interest and knowledge related to these plants. The long-term deep cultural embeddedness of the studied species could be capitalized on to obtain a stronger societal support for the idea of arable weed species conservation. More generally, ethnobotanical and cultural embeddedness should be considered more seriously when efforts and instruments for the conservation of arable weed communities are designed.

## Figures and Tables

**Table 4:** Metaphorical connotations of the studied wildflowers symbolizing human characters and emotions (own synthesis based on the cultural uses presented in Section 3.5.1, Section 3.5.2, Section 3.5.3, Section 3.5.4, Section 3.5.5, Section 3.5.6 of this article).

Character, Emotion Being Symbolised	Species
Homesickness, nostalgia, bucolic reminiscence	*P. rhoeas, C. cyanus, D. consolida*
Patriotism	*P. rhoeas, C. cyanus, D. consolida*
Historical remembrance	*P. rhoeas, C. cyanus*
Passion, infatuation, burning love, lovesickness	*P. rhoeas*
Impermanence, transience, ephemerality, fragility	*P. rhoeas*
Purity, innocence, virginity	*C. cyanus*
Pertinence, faithfulness, loyalty	*C. cyanus*
Gentle and tender love	*C. cyanus*

## Data Availability

Not applicable.
